# Reductive Upgrading of Biomass-Based Levulinic Acid to γ-Valerolactone Over Ru-Based Single-Atom Catalysts

**DOI:** 10.3389/fchem.2022.895198

**Published:** 2022-04-01

**Authors:** Ye Meng, Yumei Jian, Dandan Chen, Jinshu Huang, Heng Zhang, Hu Li

**Affiliations:** State Key Laboratory Breeding Base of Green Pesticide and Agricultural Bioengineering, Key Laboratory of Green Pesticide and Agricultural Bioengineering, Ministry of Education, State-Local Joint Laboratory for Comprehensive Utilization of Biomass, Center for R&D of Fine Chemicals, Guizhou University, Guiyang, China

**Keywords:** biomass, value-added chemicals, γ-Valerolactone, single-atom catalysts (SACs), levulinic acid (LA)

## Abstract

With the great adjustment of world industrialization and the continuous improvement of energy consumption requirements, the selective conversion of biomass-based platform molecules to high-value chemicals and biofuels has become one of the most important topics of current research. Catalysis is an essential approach to achieve energy-chemical conversion through the “bond breaking-bond formation” principle, which opens a broad world for the energy sector. Single-atom catalysts (SACs) are a new frontier in the field of catalysis in recent years, and exciting achievements have been made in biomass energy chemistry. This mini-review focuses on catalytic conversion of biomass-based levulinic acid (LA) to γ-valerolactone (GVL) over SACs. The current challenges and future development directions of SACs-mediated catalytic upgrading of biomass-based LA to produce value-added GVL, and the preparation and characterization of SACs are analyzed and summarized, aiming to provide theoretical guidance for further development of this emerging field.

## Introduction

With advances in research and industrial technology, biomass is now increasingly deemed as one of the most valuable renewable resources that can be converted into a variety of platform molecules, fine chemicals, biofuels, and solid biochars (Li et al., 2015; Liu et al., 2015; Li et al., 2017; Li H et al., 2019; Li H et al., 2020; Meng and Li, 2021; Meng et al., 2022). γ-Valerolactone (GVL) is an important platform molecule and green solvent, which can be used to produce liquid fuels, polymers, intermediates for fine chemicals synthesis, and seasonings (Wright and Palkovits, 2012; Zhang, 2016; Ye et al., 2020). Catalytic hydrogenation of biomass-based levulinic acid (LA) is one of the most effective methods to produce GVL, which has attracted increasing attention in recent years. Homogeneous catalysts can be uniformly dispersed in the catalytic system, which can effectively promote LA hydrogenation to produce GVL. However, due to the high boiling point of GVL (207–208°C), the product/catalyst separated by distillation is not economical, resulting in a homogeneous system not suitable for the target production of GVL (Zhang, 2016). Therefore, the large-scale production of GVL almost certainly depends on heterogeneous catalysts, because heterogeneous catalysts are easier to be separated from nonvolatile GVL (Zhang, 2016; Zhang et al., 2019).

Metal sites play an important role in the hydrogenation, intramolecular (trans) esterification or dehydration of LA to obtain GVL (Dutta et al., 2019). Therefore, improving the utilization rate of metal atoms is the key step to reduce the production cost and obtain high efficiency in the whole catalytic process. Single-atom catalysts (SACs) are supported catalysts containing only isolated active centers (Chen et al., 2018; Ji et al., 2020), in which a strong interaction or considerable charge transfer between highly dispersed single metal atoms and solid supports. This unique structure allows single-metal atoms of the SACs to have a desired electronic structure and carry specific electrical charges different from those of conventional metal nanoparticles (Ding et al., 2019; Xue et al., 2022). It seems one of the most promising materials for rational utilization of metal resources and atomic economy. In theory, all the involved metal atoms could behave as homogeneously as homogeneous catalysts, with an atomic efficiency of 100% (Ding et al., 2019). SACs have shown excellent catalytic performance, with respect to activity, selectivity, and stability, in catalytic conversion of various biomass-derived feedstocks into target value-added chemicals (De et al., 2022).

Some excellent reviews have depicted the catalytic production of GVL from biomass-based platform molecule LA in different perspectives (Wright and Palkovits, 2012; Zhang, 2016; Ye et al., 2020), while SACs applied in this field have not been comprehensively discussed till now. This mini-review focuses on the preparation and characterization of SACs and their application in the conversion of LA to GVL. The advantages and disadvantages of SACs, and the catalytic activity of LA over SACs are introduced emphatically.

## Catalytic Conversion of LA to GVL

**FIGURE 1:**
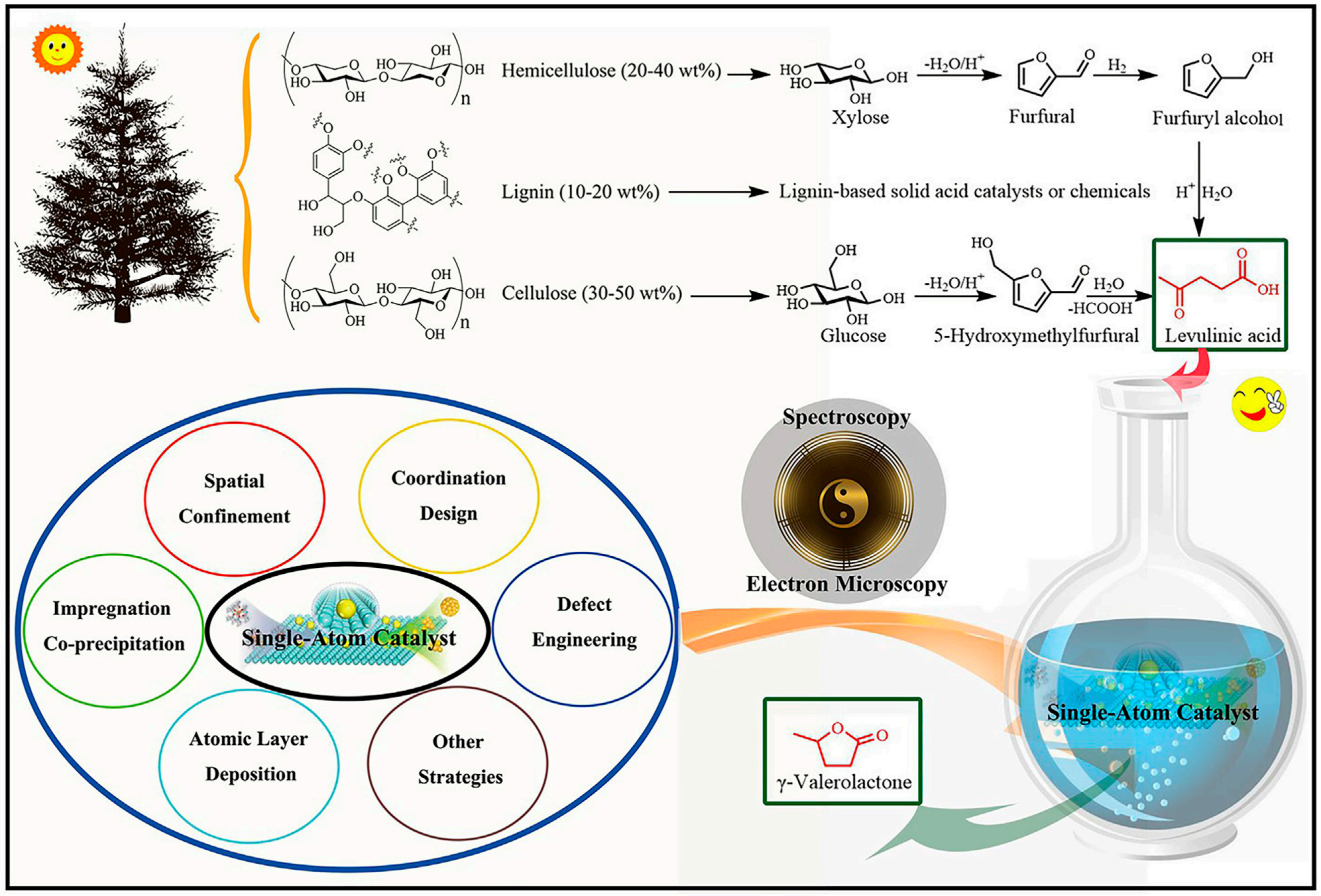
The schematic of reductive upgrading of biomass-based LA (levulinic acid) to GVL (γ-valerolactone) over single-atom catalysts.

LA is an important platform molecule, which can be easily and economically produced from lignocellulose *via* a simple and high-yield acid-catalyzed hydrolysis process (Figure 1) (Rackemann and Doherty, 2011; Ruppert et al., 2012; Khan et al., 2018). The condensation ability of LA is related to hydrogenation, and the generation of GVL may be realized through the hydrogenation of unsaturated carbon-carbon or carbon-oxygen bonds. Therefore, GVL can be synthesized by hydrogenation of LA through following two reaction mechanisms. *1*) Hydrogenation of ketone group of LA to produce unstable intermediate 4-hydroxy-pentanoic acid (HPA), and subsequent dehydration followed by an intramolecular esterification, resulting in the ring closure to yield GVL. *2*) LA is directly dehydrated to produce α-angelica lactone and then hydrogenated to produce GVL (Zhang, 2016; Dutta et al., 2019; Ye et al., 2020). In these two pathways, the hydrogenation step depends on the metal active sites of the catalyst, and the dehydration and cyclization steps are affected by the acidic conditions of the reaction system (Galletti et al., 2012; Lin et al., 2018; Cai et al., 2019). In this regard, improving the utilization rate of metal atoms is a key step to reducing the cost and obtaining high efficiency for the overall catalytic process.

In recent years, SACs have opened up a vast field of catalysis because of their advantages of high atomic utilization rate, high activity, high stability, and high selectivity. Many different active phases, including noble and non-noble metals, have been tested to catalyze the conversion of LA to GVL (Yan et al., 2013; Yuan et al., 2013; Mai et al., 2014). Ruthenium-based catalysts remain preferred because they are usually the most active and selective catalysts. Using metal-organic framework (MOF) NH_2_-MIL-125 as the precursor, Zhang et al. prepared Ru single-atom catalyst (Ru/TiO_2_@CN) by loading anatase and rutile mixed-phase TiO_2_ on nitrogen-doped amorphous carbon through high-temperature calcination (Zhang et al., 2021). At room temperature, the conversion of LA and selectivity of GVL could reach 100% through hydrogenation and dehydration reactions. The turnover frequency (TOF) of the catalyst was about 35 times higher than that of the industrial Ru/C catalyst, also with superior selectivity toward GVL. In addition, the mechanism study showed that LA is first converted to HPA through the hydrogenation process, and then HPA is rapidly dehydrated to GVL. The stability of 0.85 wt% Ru/ZrO_2_@C catalyst prepared by wet impregnation method for the conversion of LA to GVL was significantly improved (Cao et al., 2017). ZrO_2_@C support, with a nano t-ZrO_2_ (3.3 nm) structure embedded in amorphous carbon, was obtained by thermal decomposition of UiO-66 material (Zr-MOF). The catalytic performance of Ru/ZrO_2_@C in LA-to-GVL conversion was tested under 10 bar H_2_ at 150°C in an aqueous solution, and compared with that of commercial 5 wt% Ru/C. Both catalysts could achieve the full conversion of LA and the quantitative yield of GVL, while 5 wt% Ru/C exhibited poor deactivation resistance after the first operation. Inductively coupled plasma optical emission spectroscopy (ICP), X-ray photoelectron spectroscopy (XPS), high-resolution transmission electron microscopy (HR-TEM), aberration-corrected scanning transmission electron microscopy (AC-STEM), temperature-programmed reduction (TPR), and physical adsorption data showed that the rapid deactivation of Ru/C was mainly due to the leaching of Ru and the loss of surface area caused by carbon deposition in micropores (Cao et al., 2017). In contrast, the self-made Ru/ZrO_2_@C catalyst could be repeatedly recycled in water and high proton aqueous solution, with no significant decrease in catalytic performance. Yang et al. reported a single-atom catalyst (SAC) with a core-shell structure synthesized by core-shell bistability strategy using amine-modified Ru_1_/Fe_3_O_4_ as the core and periodic mesoporous organic silicon (PMO) as the shell (Yang et al., 2021). The Ru atom (0.76 wt%) was inserted into the oxygen vacancy of Fe_3_O_4_ spheres and stabilized by the amine group of 1,6-hexanediamine. Hollow PMO spheres are hydrophobic, which provides a strong barrier for the core of internal Ru_1_/Fe_3_O_4_, while the mesopores of the shell (4.2 nm) together with the cavity enhance the porosity of the catalyst. The conversion rate of LA was up to 99% with the GVL selectivity of up to 98.9%, while the high catalyst stability was still maintained after seven cycles. Regulating the electronic structure of metal catalysts can improve the catalyst performance, but it is very challenging work to regulate the electronic structure of atom-level catalysts. Fortunately, a RuCo single-atom alloy (SAA) catalyst was recently reported, in which precisely tailored electron-rich Ru atoms were confined to Co lattices, and ZIF-67 containing Ru was prepared by pyrolysis (Shao et al., 2021). The experimental study and calculation simulation showed that its activity was derived from the intrinsic active site of RuCo SAA. It was further illustrated that the electron-rich Ru single atom promoted the adsorption of C=O/H_2_ and the dissociation of H_2_ to H atom, especially beneficial to hydrogenation of the unsaturated γ-C of LA, which is the rate-determining step of LA hydrogenation. The more Ru content, the better the reaction activity of RuCo is for LA-to-GVL, and the TOF value could reach 3500 h^−1^, which is 27 times higher than that of commercial 5 wt% Ru/C catalyst. Han et al. also reported a strategy using notched polyoxomtalate (N-POM) to immobilize Ru atoms, which could prevent the aggregation of Ru during pyrolysis and obtain atom-dispersed Ru catalysts anchored onto uniform subsupporter WO_*x*_ clusters on carbon-nitrogen (Ru_1_@WO_*x*_/CN) (Han et al., 2021). The synthesized Ru_1_@WO_*x*_/CN catalyst had good catalytic activity for the hydrogenation of LA to GVL under solvent-free conditions (99% conversion, and 100% selectivity). Properly regulating the active center of the Ru electronic structure can promote the formation and dehydration of HPA intermediates to form GVL. The N-POM strategy is also excellent in preparing a series of atom-dispersed noble metal atoms, which provides an opportunity to find SACs. The stability of the catalyst in polar solvents is also a very important indicator in the catalytic upgrading of the biomass platform molecules. The activity, selectivity, and stability of Ru-based catalysts supported on TiO_2_, ZrO_2_, and C in the conversion of GVL from LA at 30 bar H_2_ and 150°C were investigated (Ftouni et al., 2016). All the tested catalysts showed good GVL yield in fresh use, but only Ru/ZrO_2_ catalyst could maintain high yield in multiple cycles. Surprisingly, the widely used Ru/TiO_2_ catalyst showed rapid deactivation after the first catalytic test. The characterization structure showed that the partial deactivation of Ru was attributed to the reduction of Ti support and the coating of Ru nanoparticles, namely the interaction of harmful strong metal supports, rather than the sintering or coking of Ru. In contrast, the Zr support showed no signs of activity reduction after five cycles and had high morphological and structural stability. It is worth noting that in the fresh Ru/ZrO_2_ catalyst, even if the Ru loading is 1 wt%, Ru can still be completely dispersed on the fresh catalyst, and some Ru nanoparticles can be observed after recycling. Further studies on Ru/ZrO_2_ catalysts showed that the dioxane was easily replaced by milder solvents, including GVL itself. Recently, in addition to the SACs constructed with Ru as the active center, which shows excellent catalytic performance in the research system of LA-to-GVL conversion, Ir-based SACs have also come into the vision of researchers. Cao et al. first reported an ultra-stable Ir-based SAC (0.6 wt% Ir@ZrO_2_@C) (Cao et al., 2019). In polar and proton reaction media (pH = 3 and pH = 1) under harsh conditions (T = 155°C, P_H2_ = 40 bar), 2.7 wt% Ir/C and 0.6 wt% Ir/ZrO_2_ nanocatalysts showed significant deactivation in recycling experiments, mainly due to the leaching of Ir into acidic reaction media. In contrast, Ir@ZrO_2_@C SAC catalyst showed advantages in selectivity and unprecedented stability toward GVL, which could be recycled continuously seven times (pH = 3) and six times (pH = 1) in aqueous solution without deactivation and metal sintering or leaching. It is thus can be observed that the *in-situ* synthesis process that limits the entry of a single atom into the metal-organic framework by space proves to be an effective method for preparing acid-resistant solid catalysts.

## Preparation and Characterization

The catalytic performance of SACs with a good structure and coordination environment is greatly improved, which is helpful for the identification of active sites and the study of catalytic mechanisms at an atomic level. Although SACs have made proud achievements in various catalytic fields such as thermal catalysis, photo-catalysis, electro-catalysis, and photoelectron-catalysis, the preparation of highly dispersed SACs still presents some challenges (Ji et al., 2020). Here, the synthesis strategies of SACs are summarized for a better understanding of the construction process of SACs to promote the application of SACs for LA-to-GVL (e.g., impregnation, co-precipitation, spatial confinement, coordination, and defect strategy) (Figure 1). As a typical preparation method, the impregnation and co-precipitation methods are of great significance for the preparation of SACs by controlling metal loadings and selecting suitable high surface area supports (Wei et al., 2014; Zhang X et al., 2018; Ye et al., 2019). For example, Ftouni et al. prepared two 1 wt% Ru catalysts supported on TiO_2_ and monoclinic ZrO_2_ by wet impregnation method (Ftouni et al., 2016). The spatial confinement strategy is to disperse single atoms in space using porous materials such as zeolites (Masoumifard et al., 2018; Liu L et al., 2020), metal-organic frameworks (MOFs) (Jiao and Jiang, 2019), and covalent organic frameworks (COFs) (Yi et al., 2018; Zhong et al., 2019). N-POM was used to confine Ru atoms to prepare highly dispersed Ru1@WO_*x*_/CN catalysts for producing GVL (Han et al., 2021). The coordination usually takes the target single atom as the coordination center, which then coordinates with the ligand containing lone pair electrons to form highly dispersed SACs. Ligands commonly used are polymers and polymer-derived materials with abundant heteroatoms (Pan et al., 2018; He et al., 2019), MOFs and their derivatives (Zhang et al., 2016; Yang et al., 2019), carbon-based materials (Li X et al., 2020), and C_3_N_4_ and graphdiyne (Tan et al., 2017). The defect strategy is to prepare materials with dispersed defect sites from relatively intact materials, followed by introducing single atoms into the defect sites to form highly dispersed SACs (Beniya and Higashi, 2019). These materials usually include oxides, hydroxides (Dvorak et al., 2016; Sun et al., 2019), graphene, and other deficiency-rich materials (Fei et al., 2019). The strategy of direct conversion of metal nanoparticles into SACs is to create suitable synthesis conditions to break the metal bond of nanoparticles and form a new bond between the single metal atom and the anchor chain of the support (Moliner et al., 2016). The “top-down” strategy is to directly convert metal block materials and metal oxide powers materials into SACs (Qu et al., 2018). Single-atom alloy strategy is dependent on the dispersion of metal single-atom sites into another metal nanomaterial (Greiner et al., 2018; Li M et al., 2019; Shao et al., 2021). Chemical etching strategies include direct etching of bulk metals and nanoparticles (Qiu et al., 2015), and indirect template-assisted etching into SACs (Han et al., 2017; Sun et al., 2018). Atomic layer deposition (ALD) technology has a good ability to control the deposition of single atom or cluster on the support, so it is one of the powerful methods to accurately prepare ideal single-atom materials (Sun et al., 2013; Zhang L et al., 2018). In addition, other preparation strategies that are not commonly used include photochemistry method (Karkas et al., 2016), electrochemistry method (Zhang et al., 2017), freezing-assisted method (Wei et al., 2017), microwave-assisted method (Bilecka and Niederberger, 2010), ball-milling method (Cui et al., 2016), ionic-liquid-assisted method (Xi and Sun, 2019), and so on.

With continuous efforts in recent years, researchers have developed a variety of effective synthesis strategies, as discussed above. In connection to this, how to reveal the isolated reactive centers and overall structural information of SACs is also a very important link. In this mini-review paper, the structural characterization of isolated reactive centers in SACs (especially the Ru-based SACs for LA-to-GVL conversion) was disclosed by using advanced technologies such as electron microscopy and spectroscopy (Figure 1) (Xue et al., 2022). Transmission electron microscopy (TEM) with the atomic resolution has been developed as an effective method to study the detailed structural information of isolated reaction centers and their interactions with supports. The additional energy dispersive X-ray (EDX) detector of scanning transmission electron microscopy (STEM) can further provide element mappings, clarify the atomic dispersion of metal atoms on supports, and further evaluate the dispersion degree of single atoms. In addition, aberration-corrected high-angle annular dark-field scanning transmission electron microscopy (AC-HAADF-STEM) can easily confirm the existence of isolated reaction centers on the supports, as long as the metal atoms exhibit a much higher atomic number than the supporting elements (Lee et al., 2019; Chen et al., 2020). Although electron microscope images provide effective information to identify the structural information of the catalysts, it should be noted that there are some limitations in its application in structural characterization. Due to the limited electron penetration ability of microscopic technology, it is difficult to observe the isolated metal atoms modified in the bulk phase or cavities, rather than the surface structure. In addition, the electron microscope can only image the local structure, and cannot provide the overall structure information of the SACs (Liu J et al., 2020). Therefore, some additional spectral methods are needed to provide supplementary data and support the existence of isolated metal sites in SACs. For example, X-ray photoelectron spectroscopy (XPS) is widely used to reveal the surface valence structure of SACs. Compared with pure metal, the obvious change of binding energy may explain the oxidation state of isolated metal atoms and exclude the existence of nanoparticles (Han et al., 2021; Shao et al., 2021). In addition, X-ray absorption spectroscopy (XAS) is one of the most commonly used and powerful tools to characterize SACs, including X-ray absorption near-edge structure (XANES) spectroscopy and the extended X-ray absorption fine structure (EXAFS) (Pan et al., 2019; Qiao et al., 2022). XANES can provide local electronic state information of the detected elements, while EXAFS can provide a high-resolution coordination environment and local geometry details of isolated metal sites. As site-specific characterization techniques, Fourier transform infrared spectroscopy (FTIR) and Raman spectroscopy are also widely used to evaluate the existence of isolated metal sites (Li et al., 2016; Fang et al., 2018), because they have obvious displacement relative to clusters or nanoparticles. The theoretical calculation also is one of the most important research methods (Han et al., 2021), state density, wave function |Ψ|^2^, and modeling can verify each other with electron microscopy and spectroscopy. Taking together, the above characterization methods are conducive to revealing the role of SACs in the LA-to-GVL conversion system, which provides an effective basis for the visualization of the system in the future.

## Conclusion and Perspectives

The over-exploitation of fossil resources has caused serious social and environmental problems, and the high-value conversion of renewable resources has gradually become a global research upsurge. This mini-review focuses on the conversion of LA to GVL over SACs, and also extends to the involved reaction mechanism (e.g., hydrogenation, cyclization, and dehydration), synthesis methods (e.g., impregnation, co-precipitation, spatial confinement, self-assembling, and defect sites), and structural characterization methods (e.g., electron microscopy, and spectroscopy) of SACs. Both SACs and traditional heterogeneous catalysts show high activity and selectivity in the catalytic conversion of LA to GVL. However, traditional heterogeneous catalysts tend to lose activity after reaction, while SACs show excellent stability. At present, the SACs in this field mainly use Ru species as the active sites. Therefore, the development of SCAs with cheap transition metals as the active sites is one of the major tasks for LA-to-GVL, which is conducive to reducing the catalyst cost and promoting the commercialization of this field. In addition, the development of cleaner and more sustainable catalytic systems for LA-to-GVL is also a hot topic for future research, such as single-atom electro- and photocatalytic systems. In conclusion, SACs have great potential in large-scale catalytic production of GVL from LA. Further development of facile preparation methods of SACs and eco-friendly catalytic processes, as well as elucidation of the single-atom active center is challenging but an unprecedented opportunity to promote the industrialization of SACs for biomass valorization.
